# Influence of Caloric Restriction on Constitutive Expression of NF-κB in an Experimental Mouse Astrocytoma

**DOI:** 10.1371/journal.pone.0018085

**Published:** 2011-03-30

**Authors:** Tiernan J. Mulrooney, Jeremy Marsh, Ivan Urits, Thomas N. Seyfried, Purna Mukherjee

**Affiliations:** 1 Tumor Biology Program, Georgetown University Medical Center, Washington, D.C., United States of America; 2 Albert Einstein College of Medicine, Bronx, New York, United States of America; 3 Biology Department, Boston College, Chestnut Hill, Massachusetts, United States of America; Virginia Commonwealth University, United States of America

## Abstract

**Background:**

Many of the current standard therapies employed for the management of primary malignant brain cancers are largely viewed as palliative, ultimately because these conventional strategies have been shown, in many instances, to decrease patient quality of life while only offering a modest increase in the length of survival. We propose that caloric restriction (CR) is an alternative metabolic therapy for brain cancer management that will not only improve survival but also reduce the morbidity associated with disease. Although we have shown that CR manages tumor growth and improves survival through multiple molecular and biochemical mechanisms, little information is known about the role that CR plays in modulating inflammation in brain tumor tissue.

**Methodology/Principal Findings:**

Phosphorylation and activation of nuclear factor κB (NF-κB) results in the transactivation of many genes including those encoding cycloxygenase-2 (COX-2) and allograft inflammatory factor-1 (AIF-1), both of which are proteins that are primarily expressed by inflammatory and malignant cancer cells. COX-2 has been shown to enhance inflammation and promote tumor cell survival in both *in vitro* and *in vivo* studies. In the current report, we demonstrate that the p65 subunit of NF-κB was expressed constitutively in the CT-2A tumor compared with contra-lateral normal brain tissue, and we also show that CR reduces (i) the phosphorylation and degree of transcriptional activation of the NF-κB-dependent genes COX-2 and AIF-1 in tumor tissue, as well as (ii) the expression of proinflammatory markers lying downstream of NF-κB in the CT-2A malignant mouse astrocytoma, [e.g. macrophage inflammatory protein-2 (MIP-2)]. On the whole, our date indicate that the NF-κB inflammatory pathway is constitutively activated in the CT-2A astrocytoma and that CR targets this pathway and inflammation.

**Conclusion:**

CR could be effective in reducing malignant brain tumor growth in part by inhibiting inflammation in the primary brain tumor.

## Introduction

Malignant astrocytomas are the most common primary brain tumor and represent a leading cause of cancer-related death in children and the elderly [1], [2], [3], [4]. Long-term progression-free survival is poor for most patients with malignant brain tumors [5], [6]. The inability to effectively manage astrocytomas has been due in part to the unique anatomical and metabolic environment of the brain that prevents the complete resection of tumor tissue and impedes the delivery of therapeutic agents.

The highly invasive and inflammatory phenotype of malignant astrocytoma cells as well as that of tumor associated lymphocytes and macrophages contribute to a breakdown of the blood brain barrier [7], [8], [9], [10], [11], mediated, in part, by the release of interleukins and cytokines that increase vascular permeability, and thus facilitate the transudation of plasma into the interstitium followed by the development of cerebral edema and increased intracranial pressure [7], [8], [9], [12], [13].

Although the glucocorticoid, dexamethasone, is currently the standard drug of choice for attempting to mitigate tumor-associated inflammation and edema [14], [15], [16] the drug has been found to produce a significant number of adverse effects including hyperglycemia—which may ultimately facilitate tumor growth, gastritis, gastrointestinal bleeding, weight-gain, Cushing's syndrome, and immuno-suppression [15] [16] [17], [18]. In light of the aforementioned, less toxic therapies are necessary to manage peri-tumoral inflammation and the sequelae of tumor cell infiltration and accompanying cerebral edema in patients with malignant astrocytoma.

To our knowledge, few studies exist that describe an alternative, non-steroid based approach for the management of the inflammatory phenotype of most malignant astrocytoma. Caloric restriction (CR), the total reduction in dietary food intake without producing deficiencies in vitamins, proteins, and other macro- or micro-nutrients for short term study, has long been proposed as an alternative therapeutic approach for managing malignant brain tumor growth, delaying disease progression, and in increasing long-term survival in mice bearing orthotopically implanted tumors [19], [20], [21] [22], [23]. In addition to multiple reports suggesting that CR is a broad-spectrum inhibitor of many metabolic processes and signaling cascades in experimental brain tumors, CR has also been shown to improve the health and increase the longevity of mice bearing a malignant astrocytoma [19], [20], [21] [22], [23].

NF-κB signaling and activation is associated with cellular proliferation, apoptosis, angiogenesis and inflammation in brain and other cancers [24], [25], [26], [27], [28]. NF-κB increases the expression of a number of anti-apoptotic molecules, while also increasing the expression of angiogenic factors and pro-inflammatory mediators [26], [27], [29], [30], [31]. Five proteins comprise the mammalian NF-κB family [32], [33]. RelA (p65), RelB, c-Rel have a transactivation domain in their C-termini. In contrast, NF-κB 1 and 2 proteins are synthesized as large precursors, p105 and p100, which generate the mature p50 and p52 subunits, respectively. The expression of constitutively activated RelA/NF-κB is associated with malignancy in astrocytomas and plays a critical role in tumor invasion [24], [34]. In an inactivated state, NF-κB is located in the cytosol complexed with the inhibitory protein IκBα [35], [36]. A variety of growth factor signals can activate IκB kinase. This induces Iκbα phosphorylation, ubiquitination, and proteosome degradation. Activated NF-κB translocates to the nucleus, binds to DNA, and then activates a number of pro-inflammatory molecules like COX-2, TNF-α, IL-6, IL-8, MMP-9 [26], [37]. COX-2 expression plays a key role in the development of edema in brain tumors [9], [38].

Despite the anti-inflammatory effects of CR on aging and other neurodegenerative disorders, little is known about the effects of CR on brain cancer inflammation. A recent report showed that CR has an anti-oxidative and anti-inflammatory vasoprotective effects in aging rats [39]. In this report, life long CR significantly improved endothelial function, attenuated vascular ROS production, inhibited NF-κB activity, and down regulated inflammatory genes. Another recent study on renal ischemia perfusion injury model in mice clearly showed that short term dietary restriction and fasting can protect tissue from ischemic damage associated with improved insulin sensitivity, reduced expression of markers of inflammation, and insulin like growth factor 1 signaling [40].

The association between chronic systemic inflammation, obesity, and cancer is well established [41], [42], [43], [44], [45]. In obese patients, CR decreases levels of pro-inflammatory molecules, while increasing levels of anti-inflammatory molecules [46], [47], [48], [49]. Circulating leptin levels were lower in obese asthma patients under alternate day fasting than in patients under continuous *ad libitum* food intake [48]. Leptin has been shown to exert pro-inflammatory actions and it is possible that anti-inflammatory effects of CR in obese patients could be through reduced leptin level [45], [48].

In addition to inflammatory neoplastic cells, glioma associated inflammatory cells also influence peri-tumoral edema formation and tumor progression [7], [8], [9], [50]. Tumor associated macrophages is a major connection between cancer and inflammation [11]. Constitutive NF-κB activation in cancer cells is often increased by microenvironment signals like pro-inflammatory cytokines expressed by infiltrating leukocytes [10], [11]. We showed that CR reduced both number and expression of CD68 positive cells and protein in CT-2A tumors. Kari and co-worker showed that CR suppressed the production of inflammatory cytokines from alveolar macrophages challenge with a gram-positive bacteria and increased its phagocytic activity [51]. Hursting and co-workers recently showed that CR could reduce macrophage infiltration into MC38 colon carcinoma [52].

In this study, we investigated for the first time the effects of CR on inflammatory biomarkers in the CT-2A malignant mouse astrocytoma. A moderate 30% CR significantly reduced COX-2 and AIF-1 expression in the CT-2A astrocytoma. In addition, NF-κB p65 phosphorylation, DNA binding was significantly less in CT-2A tumors from mice receiving CR than in mice receiving the standard chow diet unrestricted. Moreover, CR reduced MIP-2 expression in tumor tissues. Lastly, CR reduced the number of macrophages in the CT-2A tumor. Our results indicate that CR has anti-inflammatory action against the CT-2A mouse astrocytoma and suggest that CR would likely be an effective anti-inflammatory dietary intervention against human brain tumors.

## Materials and Methods

### Animals and Experimental Astrocytoma

Mice of the C57BL/6J (B6) strain were obtained from the Jackson laboratory (Bar Harbor, ME, USA) and were propagated in the Boston College Animal Care Facility as previously described [53]. Adult male mice (8–10 weeks of age) were used for the studies and were housed individually in plastic cages with filter tops containing Sani-chip bedding (P.J. Murphey Forest Products Corp., Montville, NJ, USA). Cotton nesting pads were provided for all mice for warmth and the room temperature was maintained at 22±1°C on a 12 h light/dark cycle. The procedures for animal use were in strict adherence to the NIH Guide for the Care and Use of Laboratory Animals and were approved by the Institutional Animal Care Committee at Boston College. The syngeneic malignant mouse astrocytoma, CT-2A, was originally produced in our laboratory by implantation of a chemical carcinogen, 20-methylcholanthrene, into the cerebral cortex of B6 mice according to the procedure described by Zimmerman [54]. The CT-2A tumor is histologically classified as a poorly differentiated highly malignant astrocytoma that grows orthotopically as a soft, noncohesive, and highly vascularized mass, that also exhibits many molecular and biochemical similarities with high grade gliomas in humans [54],[55].

### Antibodies and Reagents

Antibodies were obtained from Cell Signaling (Beverly, MA, USA) against phospho-NF-κB (p65) (S-536), total NF-κB (p65), phospho-IκB (T-19/S-23), total IκB, COX-2, TNF-α, AIF-1, MIP-2, and for the lysis buffer. CD68 (M-20), Anti-β-actin, goat anti-rabbit, IgG-HRP, and goat anti-mouse IgG-HRP antibodies were obtained from Santa Cruz Biotechnology Inc. (Santa Cruz, CA, USA). Histone 2B antibody was obtained from Imgenex (Cambridge, MA, USA). The Nuclear extraction kit and the electrophoretic mobility shift assay (EMSA) kit were purchased from Panomics (Fremont, CA). 2,2,2-tribromoethanol and tert-amyl alcohol were obtained from Sigma (St. Louis, MO, USA). The StanBio Enzymatic Glucose Assay kit (1075-102) was obtained from StanBio Laboratories (Boerne, TX, USA). The DC Protein Assay kit was purchased from Bio-Rad (Hercules, CA, USA).

### Intracerebral (i.c.) Tumor Implantation

The CT-2A tumor is maintained in vivo by implanting an ∼1 mm^3^ fragment of tumor tissue into the right cerebral cortex of B6 mice using a trocar (size 1 mm^3^ diameter) as we described previously [56]. Briefly, mice were anesthetized with 2,2,2- tribromoethanol, i.p., and their heads were shaved and swabbed with 70% ethanol under sterile conditions. Small tumor fragments (∼1 mm^3^, estimated using 1 mm×1 mm grid) from a donor mouse were implanted into the right cerebral cortex of anesthetized recipient mice. All of the mice recovered from the surgical procedure and were returned to their cages when fully active. Initiation of tumors from intact tumor fragments is preferable to initiation from cultured cells because the fragments contain an already established microenvironment that facilitates rapid tumor growth [22], [56].

### Dietary regimens, body weight, and food intake measurements

All mice received PROLAB RMH 3000 chow diet (Lab Diet, Richmond, IN, USA) prior to the initiation of the study. This regimen contained a balance of mouse nutritional ingredients and delivers 4.1 kcal g-1 gross energy, where fat, carbohydrate, protein, and fiber comprised 55, 520, 225, and 45 g kg^−1^ of the diet, respectively. Mice were separated into individual cages 1 wk before tumor implantation, during which time body weight and food intake measurements were recorded every 3 days to gather baseline information as we described [19], [20], [21]. All tumor-bearing mice were fed *ad libitum* (AL) for the first 48 hrs after tumor implantation and were then randomly assigned to one of two diet groups that received rodent chow in either unrestricted (AL; n = 10; 5 each for growth and tissue analysis) or calorically restricted (CR; n = 10) amounts. The two groups were matched for body weight (∼28.0 g) prior to the initiation of CR. The CR group received a feeding regimen of 30% less or 70% of food AL mice daily consume. Body weight and food intake of all mice was recorded daily (1:00PM – 3:00PM). The food intake for the AL-fed mice was determined daily by subtracting the weight of the food pellets remaining in the food hopper from the amount that was provided 24 hrs earlier. New food was provided every four days for AL mice. For mice in the CR group, food pellets were dropped directly into each cage daily for easy access. Water was provided *ad libitum* for all mice. The study was continued for 15 days after tumor implantation until the AL mice started decreasing their body weight and food intake.

### Tissue processing and blood collection

All food was removed from the cages 3 hours before the blood collection. Mice were anesthetized with isoflurane (Halocarbon Laboratories, River Edge, NJ, USA) and euthanized by exsanguination, involving collection of blood from the retro-orbital sinus into heparinized Eppendorf tubes using a glass capillary tube. Whole blood was centrifuged at 3,000×g for 10 min, the plasma supernatant was collected, and aliquots (two fractions per aliquot of plasma supernatant) were stored at −80°C prior to analysis. Tumors were dissected from normal appearing brain parenchyma and immediately placed on dry ice to reduce tissue degradation. Residual normal appearing contra-lateral (left) brain tissue was also removed and frozen on dry ice [19], [22]. For immunohistochemistry tumor and brain tissues were fixed in formalin. If necessary, mice were euthanized prior to the completion of the study when brain tumors became peripherally ulcerated or when mice showed morbidity as defined by our Institutional Animal Care and Use Committee guidelines.

### Measurement of plasma glucose and plasma β-OHB

Plasma glucose and β-OHB concentrations were measured spectrophotometrically using the StanBio® Enzymatic Glucose Assay (1075-102) (StanBio Laboratory, Boerne, TX, USA) and a modification of the Williamson *et al*., enzymatic procedure [57], respectively. For blood ketone body analysis, we measured only β-OHB levels because this is the major blood ketone body in plasma [58].

### Electrophoretic Mobility Shift Assay (EMSA)

Nuclear and cytosolic extracts from AL and CR tumor tissue were prepared as described in the manufacturer's protocol (Nuclear extraction kit, Panomics, Fremont, CA). Protein concentrations were determined using the BioRad DC assay. 5 µg of nuclear extracts from tissues were mixed with biotin labeled NF-κB probes (EMSA kit; Panomics) and incubated at 15°C for 30 min in a thermal cycler. For control assays, excess unlabeled double-stranded DNA was added in the incubation mixture for DNA protein binding competition assay during the hybridization period. The mixture was then loaded on a 6% non-denaturing polyacrylamide gel and electrophoresed at 120 V in 0.5% Tris borate EDTA onto a nylon membrane at 300 mA for 30 min. After transfer, the sample was fixed on the membrane by UV crosslinking for 3 min. The bands were visualized after exposure to chemiluminescence film based on the streptavidin horseradish peroxidase (HRP) reaction.

### Western Blot Analysis of phospho-NF-κB (p65) (S-536), total NF-κB (p65), phospho-IκB (T-19/S-23), total IκB, COX-2, TNF-α, AIF-1, and MIP-2 in CT-2A under AL-fed and CR- fed conditions

CT-2A tumor and contralateral normal brain tissue were homogenized in ice-cold lysis buffer (Cell Signaling Technology, Beverly, MA, USA) containing 20 mM Tris-HCl (pH 7.5), 150 mM NaCl, 1 mM Na_2_EDTA, 1 mM EGTA, 1% Triton, 2.5 mM NaPPi, 1 mM α-glycerophosphate, 1 mM Na_3_PO_4_, 1 µg/mL leupeptin and 1 mM phenylmethylsufonyl fluoride. Lysates were transferred to 1.7 mL Eppendorf tubes, mixed on a rocker for 1 h at 4°C, and then centrifuged at 8,100×g for 20 min. Supernatants were collected and protein concentrations were estimated using the Bio-Rad DC protein assay (Bio-Rad, Hercules, CA). Approximately 10–40 µg of total protein from each tissue sample were denatured with SDS-PAGE sample buffer [63 mM Tris-HCl (pH 6.8), 10% glycerol, 2% SDS, 0.0025% bromophenol blue, and 5% 2-mercaptoethanol] and were resolved by SDS-PAGE on 4–12% Bis-Tris gels (Invitrogen). Proteins were transferred to a polyvinyl difluoride (PVDF) immobilon TM-P membrane (Millipore) overnight at 4°C and blocked in either 5% non-fat powdered milk or 5% BSA in Tris-buffered saline with Tween 20 (pH 7.6) for 1–3 hr at room temperature. Membranes were probed with primary antibodies overnight at 4°C with gentle shaking. The blots were then incubated with the appropriate secondary antibody for 1 hr at room temperature (∼22°C), and bands were visualized with enhanced chemiluminescence (Pierce, Rockford, IL, USA). Each membrane was stripped and reprobed for β-actin as an internal loading control, and the ratio of the indicated protein to β-actin was analyzed by scanning densitometry (FluorChem 8900 Software; Alpha Innotech Co., San Leandro, CA).

Aliquots of nuclear (for NFκB (p65) and cytoplasmic protein fraction (15 µg) were loaded onto 12% SDS-polyacrylamide gels for electrophoresis. Following transfer of the protein loaded SDS gel onto polyvinyl difluoride (PVDF) membrane and blocking in 5% nonfat drymilk pIκB (1∶2000), pNF-κB (1∶3000), COX-2 (1∶3000), AIF-1 (1∶1000), MIP-2 (1∶1000) and actin (1∶ 4000) were detected with primary antibodies followed by horseradish peroxidase conjugated secondary antibodies (1∶ 6000). Detection was performed using chemiluminescence (ECL Western Blotting analysis system; Amersham Bioscience) [22].

### Enzyme Linked Immunosorbent Assay (ELISA) for MIP-2

Concentrations of MIP-2 in the tumor lysates and in plasma were determined by ELISA using commercially available kit (R&D, Mouse MIP-2). Recombinant mouse MIP-2 in a buffered protein base was used as a positive control and standards as supplied in the kit. MIP-2 concentrations were measured based on protein concentration in lysates and plasma.

### Immunohistochemistry for NF-kB and CD-68 staining

Immunohistochemistry was performed in paraffinized tissue sections using methods as we previously described [19], [20]. Briefly, after deparaffinization the tissue sections analyzed for CD68 and NF-κB were placed in 10 mM sodium citrate buffer, pH 6.0, at 95°C for 30 min. For NF-κB antigen unmasking tissues were digested with trypsin at 37°C for 30 minutes. The sections were quenched with 0.3% H_2_O_2_ methanol for 30 min and then blocked with 10% normal goat serum in PBS. The sections were then treated with CD-68 (1∶50) or NF-κB (1∶100) (SantaCruz Biotechnology, Santa Cruz, CA) for overnight incubation at 4°C. The sections were treated with secondary antibody for 30 minutes at room temp. The sections were next treated with avidin biotin complex, followed by a DAB substrate stain according to the manufacturer's directions (Vectastain Elite ABC kit; Vector, Burlingame, CA). The sections were rinsed three times with PBS and counterstained with hematoxylin and mounted. A corresponding tissue section without primary antibody served as the negative control.

### Statistical Analysis

Body weight, food intake, tumor growth, and plasma metabolite levels were analyzed by two-tailed *t*-test to calculate a pair-wise comparison among the AL and CR groups (SPSS 14.0, Chicago, IL). In each figure, error bars are mean ± S.E.M. Western blot and EMSA analyses represent semi-quantitative estimates of the amount of the indicated protein normalized to the appropriate corresponding loading control, or protein bound to marker DNA promoter that is present in a tissue extract. The former was taken into account in the statistical evaluation of the data.

## Results

### Constitutive Expression and phosphorylation of NF-κB (p65) in the CT-2A astrocytoma and in contra-lateral normal brain as determined by Western blot analysis

**Figure 1:**
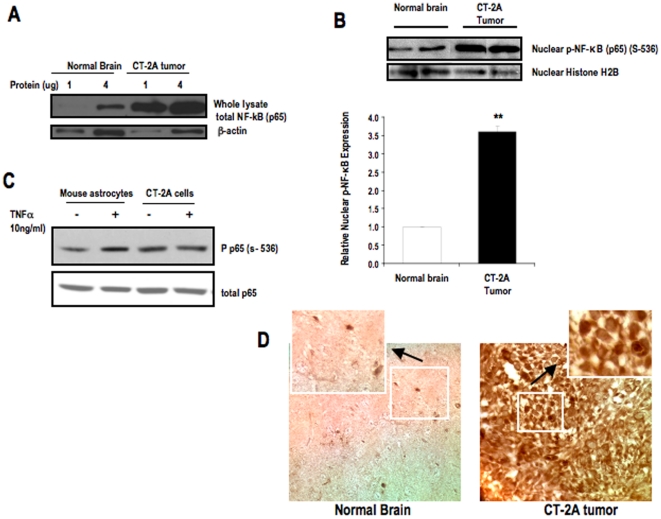
Expression and Phosphorylation of NF-κB (p65) in CT-2A astrocytoma. Western blot analysis of (**A**) Total protein expression of NF-κB (p65) in the whole lysates of tumor tissue and normal brain parenchyma. 1 and 4 µg of protein was loaded for each sample of tumor and brain tissue. (**B**) Phosphorylated NF-κB (p65) in nuclear extracts of tumor and normal brain tissues. The histogram illustrates the average relative expression of p-NFκB (p65) (S-536) to histone in nuclear extracts of the indicated tissue. Values are expressed as normalized means ± S.E.M of 4–5 independent tissue samples/group for both **A** and **B**. The asterisks in indicate that the value is significantly higher in the CT-2A astrocytoma than in contra-lateral normal brain at ** P<0.01 (Student *t*-test). Two representative samples are shown for each tissue type. (**C**) Phosphorylation of NF-κB in CT-2A cells and control mouse astrocytes in absence and presence of TNFα (10 ng/ml). (**D**) NF-κB immunostaining in CT-2A tumor and contra-lateral normal brain tissues. 3 independent mouse brain tumors were analyzed.

Western blot analysis was performed on whole lysate of CT-2A tumor and contra-lateral normal brain to examine the constitutive expression of total NF-κB (p65) in tumors. 1 and 4 µg of total protein from each of tumor and normal brain whole tissue lysate was loaded in the gel. Beta actin was used as a loading control. The results show that CT-2A tumor has constitutive expression of NF-κB (p65) when compared with normal brain (**Fig. 1A**). We showed that phosphorylated NF-κB (p65) (S-536) was significantly higher in the nuclear extract of CT-2A astrocytoma compared with that of normal brain (**Fig. 1B**). Histone was used as a nuclear loading control. The ratio of phosphorylated NF-κB to histone was significantly greater in CT-2A tumor than in normal brain. Overall the results show that both expression and activation of NF-κB (p65) (S-536) was significantly greater in the CT-2A astrocytoma compared with that of contra-lateral normal brain (**fig. 1** **A and B**). These data are consistent with recent evidence that NF-κB (p65) is constitutively overexpressed and phosphorylated in malignant astrocytomas, and that expression levels were significantly low in normal brain tissue [59]. We showed that NF-κB activation level in CT-2A cells is similar in presence or absence of TNFα whereas in control mouse astrocytes 10 ng/ml TNFα is necessary to significantly phosphorylate NF-κB at ser-536 (**fig.1C**). These findings suggest that NF-κB is present in both mouse astrocytes and CT-2A cells but it is constitutively activated in CT-2A cells but not in astrocytes. We also showed that strong immunostaining of NF-κB in CT-2A tumors whereas normal brain has les and weak NF-κB positive stained cells (**fig. 1D**). These results confirm that significantly stronger expression of NF-κB in both CT-2A tumor and cells compared to normal brain or mouse astrocytes.

### CR reduces intracerebral tumor growth, bodyweight, and plasma glucose and increases β-hydroxybutyrate levels in CT-2A-bearing mice

**Figure 2:**
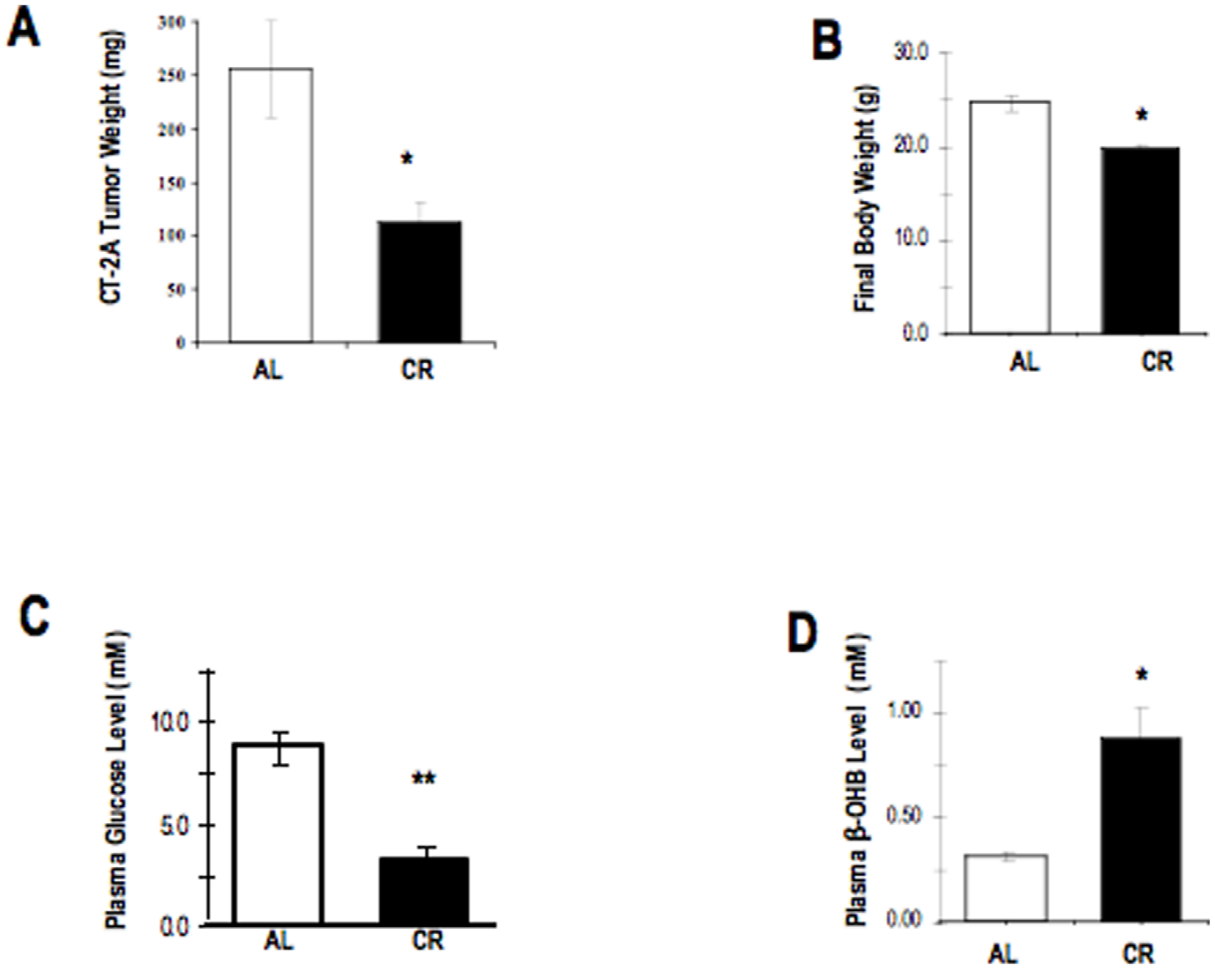
Influence of CR on tumor growth, body weight, and plasma biomarkers. Tumors were implanted i.c. on day 0. All tumor-bearing mice were fed *ad libitum* (AL) for the first 48 hrs after tumor implantation and were then randomly assigned to one of two diet groups that received rodent chow in either unrestricted (AL; n = 4) or calorically restricted (CR; n = 5) amounts. The two groups were matched for body weight (∼28.0 g) prior to the initiation of CR. The CR group received a feeding regimen of 30% less or 70% of food of AL mice consume daily. All mice were sacrificed 14–15 days after tumor implantation, as described above in the **Materials and Methods** **section**. Final weights of the mice were obtained immediately prior to sacrifice (**B**). The asterisk in indicate that the tumor growth (A), body weight (B), and glucose level (C) is significantly reduced and ketone level (D) is significantly increased in the CR group compared with the AL group at * P<0.05, ** P<0.001 (Student *t*-test).

CT-2A tumor weight was about 65% less in the CR group than in the AL group (p<0.01) (**Fig. 2A**). This is consistent with our previous findings on influence of CR on CT-2A tumor growth [19], [20]. As we previously described tumors were implanted i.c. on day 0. All tumor-bearing mice were fed *ad libitum* (AL) for the first 48 hrs after tumor implantation and were then randomly assigned to one of two diet groups that received rodent chow in either unrestricted (AL; n = 4) or calorically restricted (CR; n = 5) amounts. The two groups were matched for body weight (∼28.0 g) prior to the initiation of CR. The CR group received a feeding regimen of 30% less or 70% of AL mice daily consumption. All mice were sacrificed 14–15 days after tumor implantation when the AL mice started decreasing body weight and food intake. Body weight of CR animals was significantly reduced by 30% compared to AL mice (**Fig. 2B**). Furthermore, CR significantly reduced plasma glucose levels by 63% (**Fig. 2C**) and elevated ketone body levels by 114% (**fig. 2D**). These finding, viewed, together consistent with prior reports that moderate CR can reduce intracerebral tumor growth in CT-2A-bearing mice, and associated with reduced circulating glucose levels together with elevated ketone body levels [23], [60]. Moreover, these data support prior studies suggesting that reduced glucose and increased ketone body levels are primary markers for caloric restriction [23], [60].

### Influence of CR on nuclear expression of phosphorylated NF-κB (p65), cytosolic expression of phosphorylated IκB , total IκB, and on DNA promoter binding activity of activated NF-κB in the CT-2A astrocytoma

**Figure 3:**
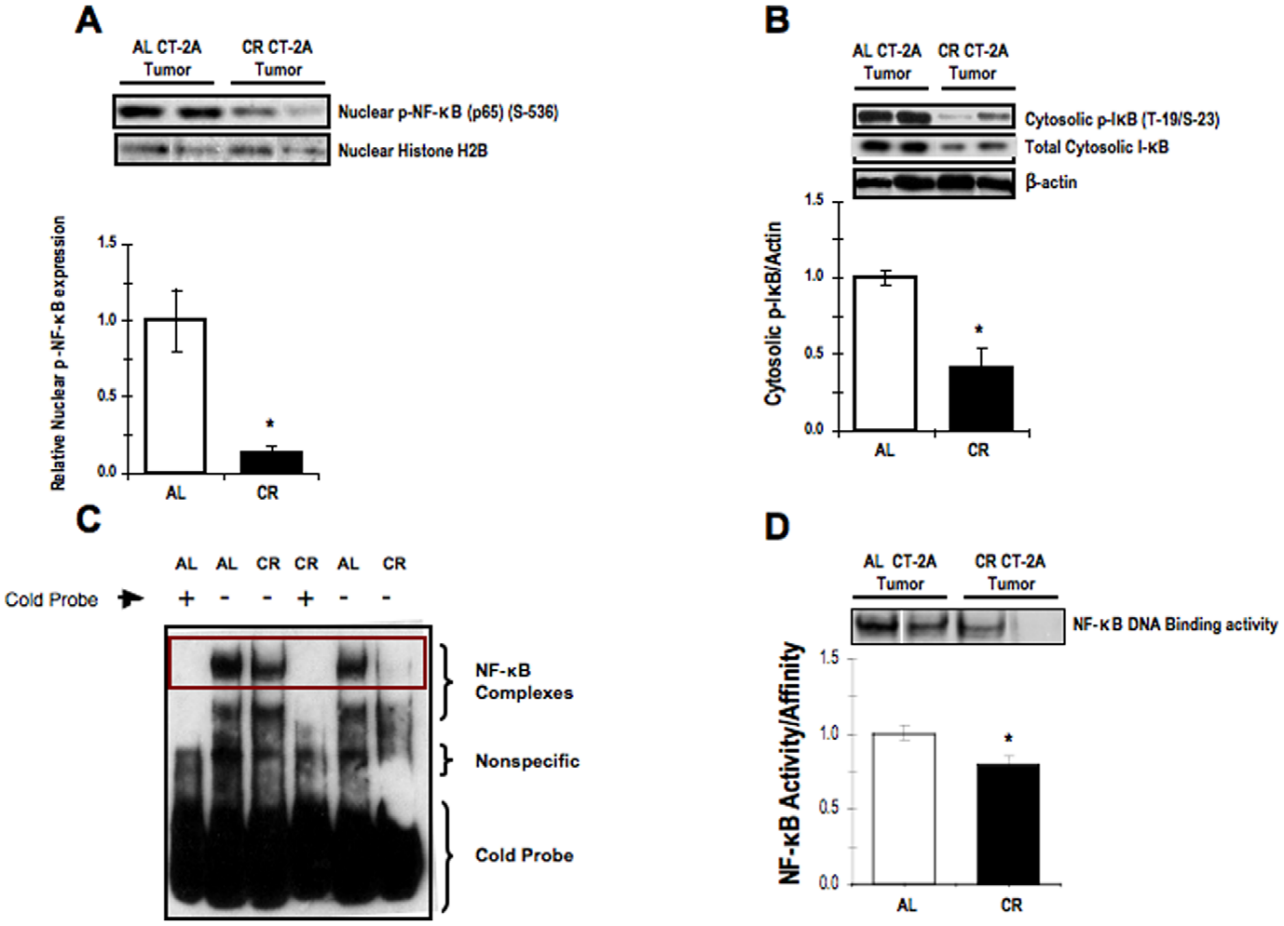
Influence of CR on NF-κB expression and activation in CT-2A astrocytoma. Nuclear expression of phosphorylated NF-κB (p65) (A); cytosolic expression of phosphorylated IκB and total lκB (B) as assessed by western blot analysis, DNA promoter binding activity of activated NF-κB in the CT-2A astrocytoma (C-D) as assessed by EMSA. The histograms illustrate the average relative expression of phosphorylated to total protein normalized to the indicated loading control in either nuclear or cytoplasmic extracts of the indicated tissue (A-B). Values are expressed as normalized means ± S.E.M of 4–5 independent tissue samples/group. The asterisks in indicate that the value is significantly different in the CT-2A astrocytoma under AL and CR condition at * P<0.05 (Student *t*-test). Two representative samples are shown for each tissue type. (**C**) Evaluation of the extent of DNA proinflammatory gene promoter binging activity by activated NF-κB in nuclear extracts of CT-2A under AL and CR condition. (**D**) Coned-down view of the DNA promoter biding activity of activated NF-κB in nuclear extracts of the NF-κB in the CT-2A astrocytoma under AL and CR condition. The histogram illustrates the average relative expression of activated NF-κB in the indicated tissue. Values are expressed as normalized means ± S.E.M of 4–5 independent tissue samples/group. The asterisks indicate that the value is significantly different in the CT-2A astrocytoma under AL and CR condition at * P<0.05 (Student *t-*test). Two representative samples are shown for each tissue type.

We showed that NF-κB overexpression is associated with activation of NF-κB (p65) in CT-2A astrocytoma. Since the phosphorylation and translocation of p65 subunit of NF-κB from cytosol to nucleus is the major component in NF-κB activation, we determined the effect of CR on activation of p65. In addition we determined the phosphorylation state of IκB, which a direct inhibitor of NF-κB activation and translocation to the nucleus where it can subsequently go on to transactivate proinflammatory gene products. More specifically, activation of the NF-κB is initiated by the signal-induced degradation of IκB proteins. Activation of enzyme I-κB kinase (IKK) in the cytoplasm phosphorylates the IκB protein, which results in ubiquitination, dissociation of IκBα from NF-κB and eventual degradation of IκBα by the proteosome, resulting in nuclear translocation of activated NF-κB that can then go on to transactivate target proinflammatory genes. The results of western blot analysis illustrate that the expression of phosphorylated NF-κB (in nuclear tissue extracts) and phosphorylated IκB (in cytosolic tissue extracts) were significantly reduced in CR CT-2A astrocytoma (**Fig. 3A-B** **, respectively**). The EMSA data showed that CR reduced the amount of binding of activated NF-κB to DNA promoters of target pro-inflammatory genes in nuclear extracts of CT-2A compared with nuclear extracts from AL-fed tumor-bearing mice (**Fig. 3C-D**). To confirm whether the DNA binding activity of NF-κB is specific, cold unlabeled DNA probe was added to the mixture of labeled probe and nuclear extract of AL tumor tissues. The cold probe competes with the labeled DNA probe for binding to the protein. The former reduced or eliminated the band's intensity (**Fig. 3C** **, lane 1**). These findings, viewed together, indicate that the CT-2A astrocytoma shows constitutive expression of NF-κB compared to normal brain parenchyma, and that CR reduces NF-κB activation and subsequent DNA binding to target promoters.

### Influence of CR on expression of the proinflammatory effectors downstream of activated NF-κB: COX-2 and AIF-1 in the CT-2A astrocytoma

**Figure 4:**
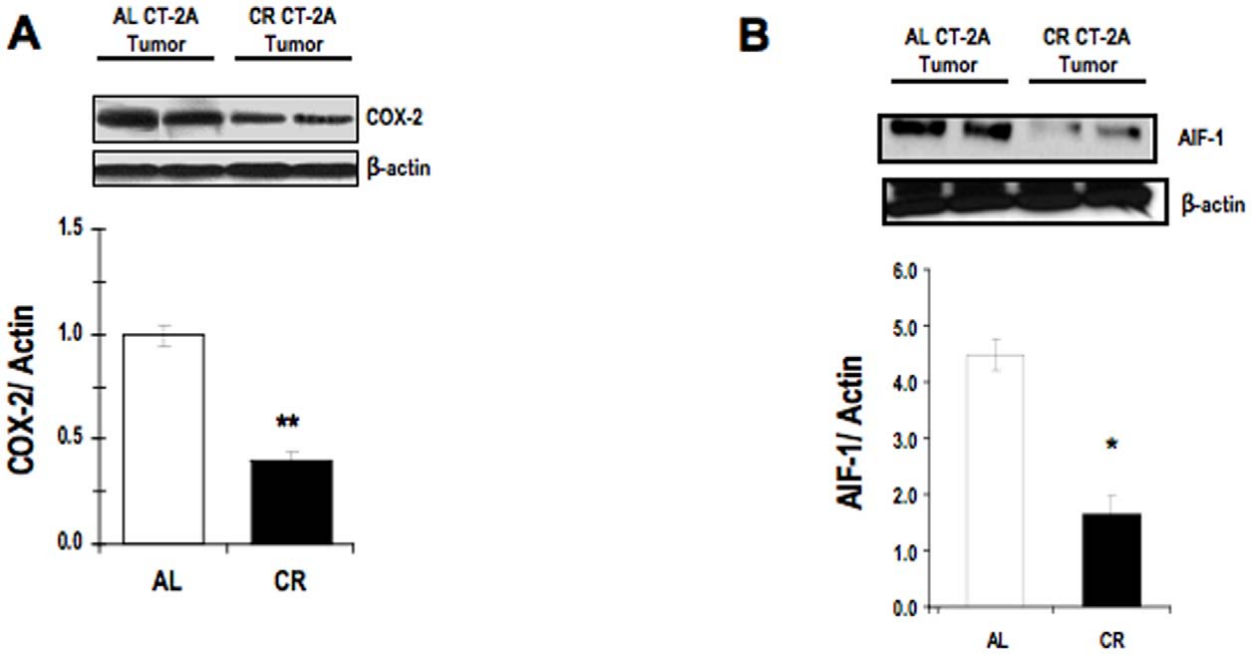
Influence of CR on inflammatory protein expressions in CT-2A astrocytoma. Cyclooxygenase-2 (COX-2) (A) and Allograft Inflammatory Factor 1 (AIF-1) (B) in cytosolic extracts of the CT-2A astrocytoma. COX-2 and AIF-1 have both been reported to be downstream proinflammatory gene-product effectors of the activated NF-κB. The histograms illustrate the average relative expression of the indicated protein normalized to β-actin in CT-2A tumors (A-B). Values are expressed as normalized means ± S.E.M of 4–5 independent tissue samples/group. The asterisks indicate that the value is significantly different in the CR tumor than in the AL tumor at * P<0.05, † P<0.001 (Student *t*-test). Two representative samples are shown for each tissue type.

In contrast to COX-1 which is constitutively expressed in nearly all tissues and mediates the synthesis of prostaglandins required for tissue homeostasis, COX-2 expression is inducible and increases in response to various stimuli including inflammatory signals, mitogens, cytokines, and growth factors, as would occur in the presence of infection, inflammation, and neoplastic processes [61]. Furthermore, COX-2 has been implicated in the growth and progression of human malignant gliomas [61]. COX-2 expression was significantly less in the tumor of CR fed mice than in that in the AL fed mice (**Fig. 4A**). Allograft inflammatory factor 1(AIF1) is a protein that encoded by the AIF1 gene in humans [62]. AIF1, also known as Iba1, is a 17 kDa protein and calcium-binding molecule involved in cellular activation and cell cycle progression [63], [64]. AIF-1 expression, which has been established as a pro-inflammatory gene product of active NF-κB, has been found in various human cells and tissues such as macrophages, thymus, liver, lung, and subtypes of invasive malignant gliomas [62], [65], [66], [67]. The results of Western blot analysis illustrate that CR significantly reduced expression of AIF-1 in total protein lysates of the CT-2A astrocytoma (**Fig. 4A-B**). We found that the basal level of COX-2 and AIF-1 in normal brain are at very minimum level (data not shown).

### Influence of CR on macrophages inflammatory protein 2 (MIP-2) concentration in the CT-2A tumor and in the plasma

**Figure 5:**
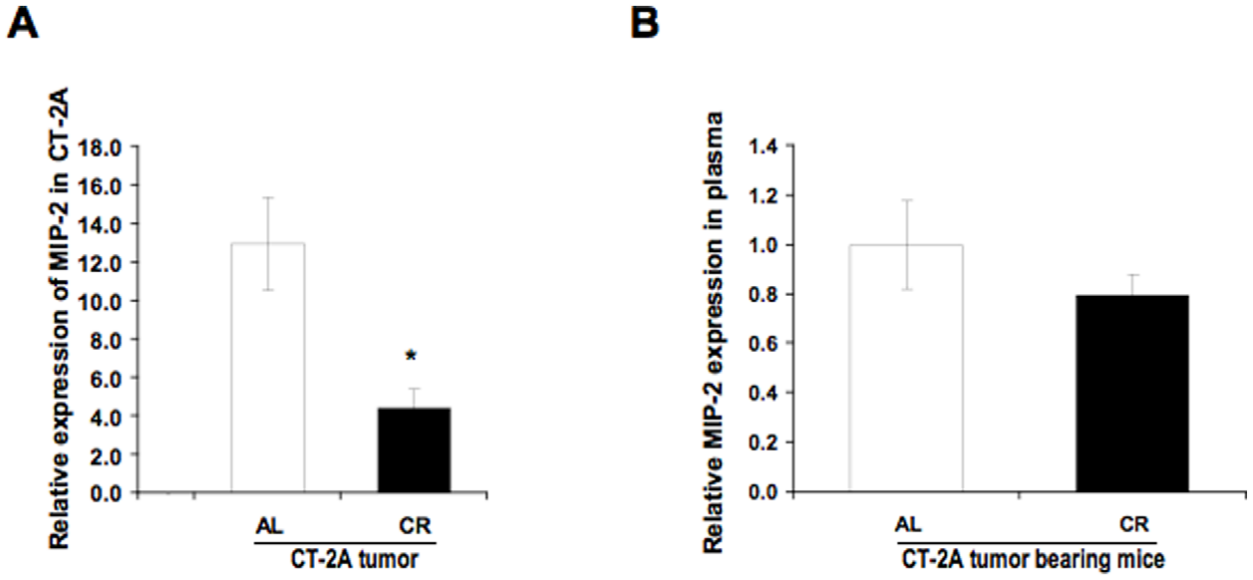
Influence of CR on the Macrophage Inflammatory Protein-2 (MIP-2) concentration. MIP-2 concentration in brain tumor lysates and in the plasma of mice bearing orthotopically implanted CT-2A tumors as assessed by ELISA. The histograms illustrate the average relative expression of the indicated protein in CT-2A from AL- and CR-fed mice (A). Influence of CR on expression of the indicated protein in the plasma of CT-2A-bearing mice fed AL or CR (B). All values are expressed as means ± S.E.M of 4-5 independent tissues or plasma samples/group. ** P<0.01 indicates that the average MIP-2 level in tumors from the AL-fed group is significantly different from that in tumors from the CR-fed group (Student *t*-test).

Macrophage inflammatory protein (MIP-2)/CXCL2 is an inducible murine chemokine involved in attraction of polymorphonuclear granulocytes to site of infection [68]. CXCL2/MIP-2 induction was observed in macrophages, epithelial cells, vascular endothelial cells, astrocytes, mast cells and neutrophils [69], [70] [71] [72]. Zampetaki et al observed that NF-κB activation induced MIP-2 gene expression in murine macrophages under hypoxic conditions [73]. Although few studies have showed that CR could reduce the MIP-2 production in inflammatory sites in murine peritonitis [74], no prior studies have determined if CR is effective in reducing MIP-2 expression levels in cancer tissue, especially in malignant astrocytoma. To this end, ELISA was performed on total protein extracts of CT-2A tumors from AL- and CR-fed brain tumor bearing mice. The results show that CR significantly reduced the protein expression of MIP-2 in CT-2A lysates compared with lysates from AL tumor (**Fig. 5A**); however, there was not a significant difference in plasma MIP-2 levels between the two groups (**Fig. 5B**). We found a significantly less amount of MIP-2 in the normal brain in ELISA (data not shown). These findings indicate that CR reduces MIP-2 levels in the CT-2A tumor compared with tumors from AL-fed tumor-bearing mice. Furthermore, these local changes in MIP-2 expression were not reflected on a systemic level, as there was no significant difference in plasma MIP-2 levels in CT-2A-bearing from the AL- and CR-fed group (P = 0.378) (**Fig. 5B**).

### Influence of CR on CD68 expression in the CT-2A tumor

**Figure 6:**
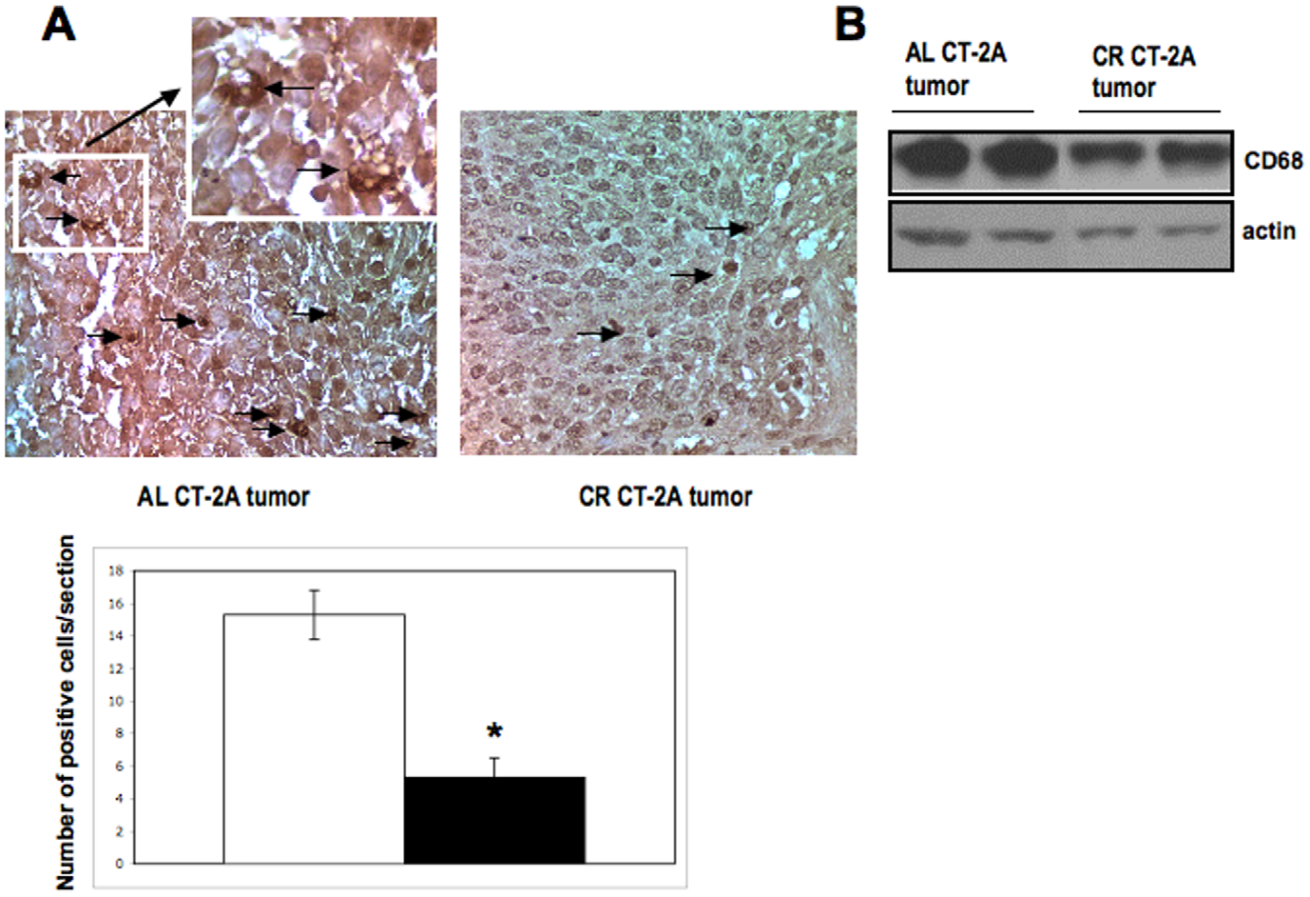
Influence of CR on CD68 expression in CT-2A tumor. Immunohistochemistry staining shows brown CD68 positive (black arrow) cells at x400 (A). Each section was representative of the entire tumor. All images are produced from digital photograph. The histograms illustrate the average number of brown cells in the entire tumor section at 400× from 3 independent mouse tumors in each group. *P<0.05 indicates that the average CD68 positive cells in tumors from the CR-fed group is significantly less from that in tumors from the AL fed groups. (B) 20 µg of tumor lysates were loaded and incubated with primary CD68 antibody. β-actin was used as loading control. 3 independent samples of each AL and CR group were used.

CD68 is a glycosylated transmembrane protein and is specifically expressed in macrophages [75]. Number of macrophages as detected by brown positive CD68 immunostaining is significantly less in CR CT-2A tumor than in AL CT-2A tumor (**Fig. 6A** **)**. The brown DAB staining intensity is much stronger in macrophages from AL tumor tissues than that in CR tumor tissues. The reduction in CD68 positive cells in CR tumor is also associated with significant reductions in the protein expression of CD68 in tumor tissues as detected by western blot (**Fig. 6B** **)**. These findings indicate that CR reduces the number of infiltrating macrophages in the brain tumor.

## Discussion

We have clearly established the role of CR in reducing experimental brain tumor growth through its influence on multiple signaling cascades that differentially influence energy metabolism in tumor tissue and normal brain parenchyma [20], [22] [21]. To our knowledge no previous studies have examined the role of CR in modulating the expression of pro-inflammatory markers in the CT-2A astrocytoma or in other tumors. Our findings suggest that the anti-tumor effects of CR can be attributed, in part, to an attenuation of localized or systemic inflammation. The present study demonstrates that CR reduces signaling through the NF-κB pathway thus contributing to the anti-inflammatory effects of CR in managing CT-2A astrocytoma growth. We found that the anti-inflammatory effects of CR in the CT-2A astrocytoma were associated with (i) reduced nuclear NF-κB activation and subsequent DNA binding to promoters of proinflammatory genes, (ii) reduced COX-2 and AIF-1 expression, (iii) reduced CD68 and MIP-2 expression.

NF-κB is a master transcription factor for pro-angiogenic and anti-apoptotic characteristics of human malignant brain tumors and NF-κB overexpression is a hallmark of GBM [24], [76]. We previously showed that the CT-2A astrocytoma is highly angiogenic with numerous thrombotic vasculatures [19], [20]. Inflammation dependent angiogenesis is known to drive tumor growth and progression [77], [78], [79]. In addition to endothelial cells and cancer cells, surrounding stromal and infiltrating cells also influence angiogenesis [10]. Persistent angiogenesis driven by myeloid inflammatory cells is now recognized in cancer progression [80]. The constitutive expression of NF-κB and together with over expression of related inflammatory molecules in the CT-2A astrocytoma indicates a high degree of inflammation. Our data show that CR significantly reduced the activation of NF-κB and downstream pro-inflammatory molecules in CT-2A tumor. Although the molecular mechanisms by which CR reduces NF-κB activation remains unclear, the anti-inflammatory effects of CR are robust. One possible mechanism could involve a CR-induced reduction of tumor necrosis factor (TNFα), which could suppress NF-κB activation in CT-2A tumor [39], [51]. Tumor associated macrophages and other immune cells are the major source of TNFα [81], [82], [83]. As we found that macrophage specific CD68 positive cells are significantly less in CR tumor than in AL tumor it is likely that pro-inflammatory cytokines release from macrophage will be less in the CR tumor tissue microenvironment. An array analysis of pro-inflammatory cytokines in separated cell population of brain tumor cells and tumor infiltrating macrophages from both AL and CR fed mice would better define the influence of CR on macrophage-induced inflammation.

A significant positive correlation has been found between glioma grade and the activation status of Akt and NF-κB [84]. Reduction of PTEN expression, as we previously showed in the CT-2A tumor, could activate NF-κB through the PI3kinase/Akt pathway [22]. CR induced inhibition of IGF-1/Akt signaling was recently demonstrated in CT-2A tumor and therefore could be a plausible mechanism of inhibiting NF-κB activation [22].

Glucose is used as a major fuel for normal brain energy metabolism, but brain cells can metabolize ketone bodies for energy when blood glucose levels decrease as in CR [85]. CR is known to reduce circulating level of glucose and to elevate the levels of ketone bodies [22], [60], [86], [87]. Recent reports suggest that ketone based metabolism reduces inflammation in epilepsy and in other neurodegenerative disorders [88]. We also recently reported that elevated ketone levels modulated GBM progression in a patient treated with a calorie restricted KD [89]. Alternate day caloric restriction in overweight adults with moderate asthma significantly increased ketone bodies in the serum and reduced oxidative stress and inflammation with improved pulmonary function in 8 weeks trial period [48]. In contrast to other studies, this alternate day caloric restriction diet had no significant effect on serum glucose and insulin level [48]. Ketone metabolism results in a decreased production of reactive oxygen species, known to contribute to inflammation [90] [85]. Further studies will be needed to determine if elevated ketones or reduced glucose underlie the anti-inflammatory effects of CR in brain tumors.

NF-κB mediated COX-2 expression is also greater in more aggressive than in less aggressive gliomas [91]. Tumor associated microglia in GBM produce high levels of prostaglandin E (PGE), which could be mediated by the expression of COX-2. [9], [92]. A selective COX-2 inhibitor appears to be as effective as dexamethasone in prolonging survival in a rat brain tumor model [38]. We found that COX-2 protein expression in CT-2A astrocytoma was significantly lower in CR fed mice than in AL fed mice. Extensive evidence suggests reduced chronic inflammation underlies the anti-aging mechanisms of CR. All these studies were carried out with a long-term CR regimen [93], [94]. However, Jung et al recently found that CR administered for only 10 days could suppress lipid peroxidase and COX-2 activities in aged rat kidney [95]. This observation is consistent with our findings that CR for 10 days was able to reduce COX-2 expression in the orthotopic CT-2A tumor. The reduced COX-2 expression may be associated with reduced NF-κB activation in CT-2A tumor from CR mice. Nevertheless, we cannot exclude the possibilities that other molecules may be involved in this process. However, further studies are required to establish this mechanistic link in *in vivo* tumor model.

AIF1/Iba1 is thought to play an important role in chronic immune inflammatory processes, especially those involving macrophages. Previous studies indicate that microglia/macrophage specific Iba1 participates with L-fimbrin to influence migration and phagocytosis [96]. It was not known if CR could reduce the AIF1/Iba1 expression in brain tumors. Our present findings support the hypothesis that AIF1 inhibition could be an indirect effect of reduced number of tumor infiltrating macrophages due to CR in CT-2A tumor. It is also important to mention that our recently developed highly invasive VM3 mouse brain tumor cells are of myeloid origin and are highly positive for AIF1/Iba1 by gene and protein expression [97]. The VM3 model represents the full spectrum of growth and invasive characteristics seen in human GBM [98]. Liu et al recently showed that AIF1 over-expression in a breast cancer cell line enhances the transcriptional activity of NF-κB [96]. In light of this observation, it is possible that the CR-induced inhibition of NF-κB expression inhibition could result in part from inhibition of AIF1 protein expression. Furthermore, the role of AIF1 in regulation of angiogenesis in certain tumors is evident [99]. The protein encoded by the AIF1 gene in humans is thought to be involved in the negative regulation of vascular smooth muscle cell growth, which could contribute to the anti-inflammatory response to vessel wall trauma. This observation could explain our recent unpublished results that CR restores the smooth muscle actin around the leaky vessels in CT-2A tumor and reduces leakiness and thrombosis.

MIP-2 is a CXC chemokine that is also produced by infiltrating inflammatory leukocytes like neutrophils or monocytes. Hypoxia triggers MIP-2 expression in murine macrophages through NF-κB activation and involves the p42/p44 and the PI3 kinase pathways [73]. It was also shown that NF-κB contributes to LPS-induced mouse MIP-2 gene expression in RAW 264.7 cells [100]. Our findings suggest that CR influences macrophage linked inflammatory molecules like AIF1 and MIP-2 in CT-2A tumors, and associated with NF-κB transcription in a direct or indirect manner. In the present study, the inhibitory effect of CR on MIP-2 level in tumor tissues may be linked with reduced number of infiltrating cells. However, CR might influence the immune cells within the tumor tissue to attenuate MIP-2 release in the circulation. This might explain, in part, the reason for the lack of any significant differences in MIP-2 level in the plasma of AL and CR mice.

The potential of CR as an alternative approach for brain tumor management have been extensively discussed [85], [89], [101], [102], [103]. CR could be an alternative therapeutic approach that exploits the differences in energy metabolism between normal brain cells and brain tumor cells [85]. We also reported that the restricted amount of high fat low carbohydrate diet is equally effective in reducing CT-2A and U87-MG brain tumor growth as total caloric restriction [23]. Therefore, we suggest, the amount of calories rather than the type of calories are more effective in managing tumor growth [23], [60], [104]. Additionally the restricted diet could be combined with specific drugs to further enhance therapeutic efficacy. In conjunction with our previous findings, this study not only establish the anti-inflammatory effects of CR in a brain tumor model, but also exemplifies the potential efficacy of CR as a broad-spectrum inhibitor of malignant astrocytomas.

Viewed collectively, our findings show the anti-inflammatory effects of CR in the CT-2A astrocytoma are associated with inhibition of the NF-κB pathway and the linked pro-inflammatory molecules. CR can be considered a powerful dietary intervention therapy for malignant brain tumors, as CR can simultaneously target tumor cells, tumor associated macrophages, and endothelial cells. Since implementation of CR is easy and without any adverse side effects CR alone or in conjunction with low dose of chemotherapy may have some preclinical efficacy as a non invasive therapy with reduced toxicity for malignant brain cancer.

## References

[1] Fisher PG, Buffler PA (2005). Malignant gliomas in 2005: where to GO from here?. Jama.

[2] Nicholson HS, Kretschmar CS, Krailo M, Bernstein M, Kadota R (2007). Phase 2 study of temozolomide in children and adolescents with recurrent central nervous system tumors: a report from the Children's Oncology Group.. Cancer.

[3] Lowry JK, Snyder JJ, Lowry PW (1998). Brain tumors in the elderly: recent trends in a Minnesota cohort study.. Arch Neurol.

[4] Jukich PJ, McCarthy BJ, Surawicz TS, Freels S, Davis FG (2001). Trends in incidence of primary brain tumors in the United States, 1985-1994.. Neuro Oncol.

[5] Smith JS, Jenkins RB (2000). Genetic alterations in adult diffuse glioma: occurrence, significance, and prognostic implications.. Front Biosci.

[6] Ohgaki H DP, Jourde B, Horstmann S, Nishikawa T, Di Patre PL (2004). Genetic pathways to glioblastoma: a population-based study.. Cancer Res.

[7] Lu S, Ahn D, Johnson G, Law M, Zagzag D (2004). Diffusion-tensor MR imaging of intracranial neoplasia and associated peritumoral edema: introduction of the tumor infiltration index.. Radiology.

[8] Ludwig HC, Feiz-Erfan I, Bockermann V, Behnke-Mursch J, Schallock K (2000). Expression of nitric oxide synthase isozymes (NOS I-III) by immunohistochemistry and DNA in situ hybridization. Correlation with macrophage presence, vascular endothelial growth factor (VEGF) and oedema volumetric data in 220 glioblastomas.. Anticancer Res.

[9] Badie B, Schartner JM, Hagar AR, Prabakaran S, Peebles TR (2003). Microglia cyclooxygenase-2 activity in experimental gliomas: possible role in cerebral edema formation.. Clin Cancer Res.

[10] Albini A, Sporn MB (2007). The tumour microenvironment as a target for chemoprevention.. Nat Rev Cancer.

[11] Mantovani A, Schioppa T, Porta C, Allavena P, Sica A (2006). Role of tumor-associated macrophages in tumor progression and invasion.. Cancer Metastasis Rev.

[12] Carlson MR, Pope WB, Horvath S, Braunstein JG, Nghiemphu P (2007). Relationship between survival and edema in malignant gliomas: role of vascular endothelial growth factor and neuronal pentraxin 2.. Clin Cancer Res.

[13] Pope WB, Sayre J, Perlina A, Villablanca JP, Mischel PS (2005). MR imaging correlates of survival in patients with high-grade gliomas.. AJNR Am J Neuroradiol.

[14] Gustafson MP, Lin Y, New KC, Bulur PA, O'Neill BP Systemic immune suppression in glioblastoma: the interplay between CD14+HLA-DRlo/neg monocytes, tumor factors, and dexamethasone.. Neuro Oncol.

[15] Derr RL, Ye X, Islas MU, Desideri S, Saudek CD (2009). Association between hyperglycemia and survival in patients with newly diagnosed glioblastoma.. J Clin Oncol.

[16] Lukins MB, Manninen PH (2005). Hyperglycemia in patients administered dexamethasone for craniotomy.. Anesth Analg.

[17] Rutz HP, Herr I (2005). Glucocorticoid administration in antiemetic therapy: Is it safe?. Cancer.

[18] Rutz HP, Hofer S, Peghini PE, Gutteck-Amsler U, Rentsch K (2005). Avoiding glucocorticoid administration in a neurooncological case.. Cancer Biol Ther.

[19] Mukherjee P, Abate LE, Seyfried TN (2004). Antiangiogenic and proapoptotic effects of dietary restriction on experimental mouse and human brain tumors.. Clin Cancer Res.

[20] Mukherjee P, El-Abbadi MM, Kasperzyk JL, Ranes MK, Seyfried TN (2002). Dietary restriction reduces angiogenesis and growth in an orthotopic mouse brain tumour model.. Br J Cancer.

[21] Mukherjee P, Mulrooney TJ, Marsh J, Blair D, Chiles TC (2008). Differential effects of energy stress on AMPK phosphorylation and apoptosis in experimental brain tumor and normal brain.. Mol Cancer.

[22] Marsh J, Mukherjee P, Seyfried TN (2008). Akt-dependent proapoptotic effects of dietary restriction on late-stage management of a phosphatase and tensin homologue/tuberous sclerosis complex 2-deficient mouse astrocytoma.. Clin Cancer Res.

[23] Zhou W, Mukherjee P, Kiebish MA, Markis WT, Mantis JG (2007). The calorically restricted ketogenic diet, an effective alternative therapy for malignant brain cancer.. Nutr Metab (Lond).

[24] Tsunoda K, Kitange G, Anda T, Shabani HK, Kaminogo M (2005). Expression of the constitutively activated RelA/NF-kappaB in human astrocytic tumors and the in vitro implication in the regulation of urokinase-type plasminogen activator, migration, and invasion.. Brain Tumor Pathol.

[25] Wang H, Cho CH. Effect of NF-κB Signaling on Apoptosis in Chronic Inflammation-Associated Carcinogenesis.. Curr Cancer Drug Targets.

[26] Atkinson GP, Nozell SE, Harrison DK, Stonecypher MS, Chen D (2009). The prolyl isomerase Pin1 regulates the NF-kappaB signaling pathway and interleukin-8 expression in glioblastoma.. Oncogene.

[27] Xie TX, Xia Z, Zhang  N, Gong W, Huang S. Constitutive NF-kappaB activity regulates the expression of VEGF and IL-8 and tumor angiogenesis of human glioblastoma.. Oncol Rep.

[28] Angileri FF, Aguennouz M, Conti A, La Torre D, Cardali S (2008). Nuclear factor-kappaB activation and differential expression of survivin and Bcl-2 in human grade 2-4 astrocytomas.. Cancer.

[29] Mancino A, Lawrence T. Nuclear factor-kappaB and tumor-associated macrophages.. Clin Cancer Res.

[30] Staudt  LM. Oncogenic Activation of NF-{kappa}B.. Cold Spring Harb Perspect Biol.

[31] Li  J, Gong LY, Song LB, Jiang LL, Liu LP Oncoprotein Bmi-1 renders apoptotic resistance to glioma cells through activation of the IKK-nuclear factor-kappaB Pathway.. Am J Pathol.

[32] Karin M, Lin A (2002). NF-kappaB at the crossroads of life and death.. Nat Immunol.

[33] Nabel GJ, Verma IM (1993). Proposed NF-kappa B/I kappa B family nomenclature.. Genes Dev.

[34] Korkolopoulou P, Levidou G, Saetta AA, El-Habr E, Eftichiadis C (2008). Expression of nuclear factor-kappaB in human astrocytomas: relation to pI kappa Ba, vascular endothelial growth factor, Cox-2, microvascular characteristics, and survival.. Hum Pathol.

[35] Karin M, Ben-Neriah Y (2000). Phosphorylation meets ubiquitination: the control of NF-[kappa]B activity.. Annu Rev Immunol.

[36] Baeuerle PA, Baltimore D (1988). I kappa B: a specific inhibitor of the NF-kappa B transcription factor.. Science.

[37] Karin M (2006). Nuclear factor-kappaB in cancer development and progression.. Nature.

[38] Portnow J, Suleman S, Grossman SA, Eller S, Carson K (2002). A cyclooxygenase-2 (COX-2) inhibitor compared with dexamethasone in a survival study of rats with intracerebral 9L gliosarcomas.. Neuro Oncol.

[39] Csiszar A, Labinskyy N, Jimenez R, Pinto JT, Ballabh P (2009). Anti-oxidative and anti-inflammatory vasoprotective effects of caloric restriction in aging: role of circulating factors and SIRT1.. Mech Ageing Dev.

[40] Mitchell JR, Verweij M, Brand K, van de Ven M, Goemaere N Short-term dietary restriction and fasting precondition against ischemia reperfusion injury in mice.. Aging Cell.

[41] McMillan DC, Sattar N, McArdle CS (2006). ABC of obesity. Obesity and cancer.. Bmj.

[42] Hursting SD, Nunez NP, Varticovski L, Vinson C (2007). The obesity-cancer link: lessons learned from a fatless mouse.. Cancer Res.

[43] Calle EE, Kaaks R (2004). Overweight, obesity and cancer: epidemiological evidence and proposed mechanisms.. Nat Rev Cancer.

[44] Jenks S Researchers explore mechanisms that may link obesity and cancer.. J Natl Cancer Inst.

[45] Dixit VD (2008). Adipose-immune interactions during obesity and caloric restriction: reciprocal mechanisms regulating immunity and health span.. J Leukoc Biol.

[46] Fenton JI, Nunez NP, Yakar S, Perkins SN, Hord NG (2009). Diet-induced adiposity alters the serum profile of inflammation in C57BL/6N mice as measured by antibody array.. Diabetes Obes Metab.

[47] Hursting SD, Smith SM, Lashinger LM, Harvey AE, Perkins SN. Calories and carcinogenesis: lessons learned from 30 years of calorie restriction research.. Carcinogenesis.

[48] Johnson JB, Summer W, Cutler RG, Martin B, Hyun DH (2007). Alternate day calorie restriction improves clinical findings and reduces markers of oxidative stress and inflammation in overweight adults with moderate asthma.. Free Radic Biol Med.

[49] Clement K, Viguerie N, Poitou C, Carette C, Pelloux V (2004). Weight loss regulates inflammation-related genes in white adipose tissue of obese subjects.. Faseb J.

[50] Seyfried TN (2001). Perspectives on brain tumor formation involving macrophages, glia, and neural stem cells.. Perspect Biol Med.

[51] Dong W, Selgrade MK, Gilmour IM, Lange RW, Park P (1998). Altered alveolar macrophage function in calorie-restricted rats.. Am J Respir Cell Mol Biol.

[52] Harvey AlisonE., GO, Nunez NomeliP., Perkins SusanN., Hursting StephenD. (2010). The anticancer effects of calorie restriction on MC38 colon tumors are associated with decreased macrophage infiltration Cancer Prevention Research 3 (Meeting Abstract Supplement), B54, January 7.

[53] Flavin HJ, Wieraszko A, Seyfried TN (1991). Enhanced aspartate release from hippocampal slices of epileptic (El) mice.. J Neurochem.

[54] Seyfried TN, El-Abbadi M, Roy ML (1992). Ganglioside distribution in murine neural tumors.. Mol Chem Neuropathol.

[55] Martinez-Murillo R, Martinez A (2007). Standardization of an orthotopic mouse brain tumor model following transplantation of CT-2A astrocytoma cells.. Histol Histopathol.

[56] Ranes MK, El-Abbadi M, Manfredi MG, Mukherjee P, Platt FM (2001). N -butyldeoxynojirimycin reduces growth and ganglioside content of experimental mouse brain tumours.. Br J Cancer.

[57] Williamson DH, Mellanby J, Krebs HA (1962). Enzymic determination of D(-)-beta-hydroxybutyric acid and acetoacetic acid in blood.. Biochem J.

[58] Krebs HA, Williamson DH, Bates MW, Page MA, Hawkins RA (1971). The role of ketone bodies in caloric homeostasis.. Adv Enzyme Reg.

[59] Hayashi S, Yamamoto M, Ueno Y, Ikeda K, Ohshima K (2001). Expression of nuclear factor-kappa B, tumor necrosis factor receptor type 1, and c-Myc in human astrocytomas.. Neurol Med Chir (Tokyo).

[60] Seyfried TN, Sanderson TM, El-Abbadi MM, McGowan R, Mukherjee P (2003). Role of glucose and ketone bodies in the metabolic control of experimental brain cancer.. Br J Cancer.

[61] Shono T, Tofilon PJ, Bruner JM, Owolabi O, Lang FF (2001). Cyclooxygenase-2 expression in human gliomas: prognostic significance and molecular correlations.. Cancer Res.

[62] Autieri MV (1996). cDNA cloning of human allograft inflammatory factor-1: tissue distribution, cytokine induction, and mRNA expression in injured rat carotid arteries.. Biochem Biophys Res Commun.

[63] Ohsawa K, Imai Y, Sasaki Y, Kohsaka S (2004). Microglia/macrophage-specific protein Iba1 binds to fimbrin and enhances its actin-bundling activity.. J Neurochem.

[64] Kimura M, Kawahito Y, Obayashi H, Ohta M, Hara H (2007). A critical role for allograft inflammatory factor-1 in the pathogenesis of rheumatoid arthritis.. J Immunol.

[65] Utans U, Arceci RJ, Yamashita Y, Russell ME (1995). Cloning and characterization of allograft inflammatory factor-1: a novel macrophage factor identified in rat cardiac allografts with chronic rejection.. J Clin Invest.

[66] Pashenkov M, Efendic S, Zhu J, Zou LP, Ostenson CG (2000). Augmented expression of daintain/allograft inflammatory factor-1 is associated with clinical disease: dynamics of daintain/allograft inflammatory factor-1 expression in spleen, peripheral nerves and sera during experimental autoimmune neuritis.. Scand J Immunol.

[67] Yang ZF, Ho DW, Lau CK, Lam CT, Lum CT (2005). Allograft inflammatory factor-1 (AIF-1) is crucial for the survival and pro-inflammatory activity of macrophages.. Int Immunol.

[68] Matzer SP, Rodel F, Strieter RM, Rollinghoff M, Beuscher HU (2004). Constitutive expression of CXCL2/MIP-2 is restricted to a Gr-1high, CD11b+, CD62Lhigh subset of bone marrow derived granulocytes.. Int Immunol.

[69] Wolpe SD, Sherry B, Juers D, Davatelis G, Yurt RW (1989). Identification and characterization of macrophage inflammatory protein 2.. Proc Natl Acad Sci U S A.

[70] Huang S, Paulauskis JD, Godleski JJ, Kobzik L (1992). Expression of macrophage inflammatory protein-2 and KC mRNA in pulmonary inflammation.. Am J Pathol.

[71] Mancardi S, Vecile E, Dusetti N, Calvo E, Stanta G (2003). Evidence of CXC, CC and C chemokine production by lymphatic endothelial cells.. Immunology.

[72] Armstrong DA, Major JA, Chudyk A, Hamilton TA (2004). Neutrophil chemoattractant genes KC and MIP-2 are expressed in different cell populations at sites of surgical injury.. J Leukoc Biol.

[73] Zampetaki A, Mitsialis SA, Pfeilschifter J, Kourembanas S (2004). Hypoxia induces macrophage inflammatory protein-2 (MIP-2) gene expression in murine macrophages via NF-kappaB: the prominent role of p42/p44 and PI3 kinase pathways.. Faseb J.

[74] Ikeda S, Saito H, Fukatsu K, Inoue T, Han I (2001). Dietary restriction impairs neutrophil exudation by reducing CD11b/CD18 expression and chemokine production.. Arch Surg.

[75] Holness CL, Simmons DL (1993). Molecular cloning of CD68, a human macrophage marker related to lysosomal glycoproteins.. Blood.

[76] Nozell S, Laver T, Moseley D, Nowoslawski L, De Vos M (2008). The ING4 tumor suppressor attenuates NF-kappaB activity at the promoters of target genes.. Mol Cell Biol.

[77] Jackson JR, Seed MP, Kircher CH, Willoughby DA, Winkler JD (1997). The codependence of angiogenesis and chronic inflammation.. Faseb J.

[78] Kobayashi H, Lin PC (2009). Angiogenesis links chronic inflammation with cancer.. Methods Mol Biol.

[79] Fiedler U, Augustin HG (2006). Angiopoietins: a link between angiogenesis and inflammation.. Trends Immunol.

[80] Lewis CE, Pollard JW (2006). Distinct role of macrophages in different tumor microenvironments.. Cancer Res.

[81] Moore RJ, Owens DM, Stamp G, Arnott C, Burke F (1999). Mice deficient in tumor necrosis factor-alpha are resistant to skin carcinogenesis.. Nat Med.

[82] Li Q, Verma IM (2002). NF-kappaB regulation in the immune system.. Nat Rev Immunol.

[83] Hagemann T, Robinson SC, Schulz M, Trumper L, Balkwill FR (2004). Enhanced invasiveness of breast cancer cell lines upon co-cultivation with macrophages is due to TNF-alpha dependent up-regulation of matrix metalloproteases.. Carcinogenesis.

[84] Wang H, Wang H, Zhang W, Huang HJ, Liao WS (2004). Analysis of the activation status of Akt, NFkappaB, and Stat3 in human diffuse gliomas.. Lab Invest.

[85] Seyfried TN, Mukherjee P (2005). Targeting energy metabolism in brain cancer: review and hypothesis.. Nutr Metab (Lond).

[86] Maalouf M, Rho JM, Mattson MP (2009). The neuroprotective properties of calorie restriction, the ketogenic diet, and ketone bodies.. Brain Res Rev.

[87] Heilbronn LK, Ravussin E (2003). Calorie restriction and aging: review of the literature and implications for studies in humans.. Am J Clin Nutr.

[88] Ruskin DN, Kawamura M, Masino SA (2009). Reduced pain and inflammation in juvenile and adult rats fed a ketogenic diet.. PLoS One.

[89] Zuccoli G, Marcello N, Pisanello A, Servadei F, Vaccaro S Metabolic management of glioblastoma multiforme using standard therapy together with a restricted ketogenic diet: Case Report.. Nutr Metab (Lond).

[90] Veech RL (2004). The therapeutic implications of ketone bodies: the effects of ketone bodies in pathological conditions: ketosis, ketogenic diet, redox states, insulin resistance, and mitochondrial metabolism.. Prostaglandins Leukot Essent Fatty Acids.

[91] Annabi B, Laflamme C, Sina A, Lachambre MP, Beliveau R (2009). A MT1-MMP/NF-kappaB signaling axis as a checkpoint controller of COX-2 expression in CD133+ U87 glioblastoma cells.. J Neuroinflammation.

[92] Temel SG, Kahveci Z (2009). Cyclooxygenase-2 expression in astrocytes and microglia in human oligodendroglioma and astrocytoma.. J Mol Histol.

[93] Chung HY, Kim HJ, Kim JW, Yu BP (2001). The inflammation hypothesis of aging: molecular modulation by calorie restriction.. Ann N Y Acad Sci.

[94] Chung HY, Sung B, Jung KJ, Zou Y, Yu BP (2006). The molecular inflammatory process in aging.. Antioxid Redox Signal.

[95] Jung KJ, Lee EK, Kim JY, Zou Y, Sung B (2009). Effect of short term calorie restriction on pro-inflammatory NF-kB and AP-1 in aged rat kidney.. Inflamm Res.

[96] Liu S, Tan WY, Chen QR, Chen XP, Fu K (2008). Daintain/AIF-1 promotes breast cancer proliferation via activation of the NF-kappaB/cyclin D1 pathway and facilitates tumor growth.. Cancer Sci.

[97] Huysentruyt LC, Mukherjee P, Banerjee D, Shelton LM, Seyfried TN (2008). Metastatic cancer cells with macrophage properties: evidence from a new murine tumor model.. Int J Cancer.

[98] Shelton LM, Mukherjee P, Huysentruyt LC, Urits I, Rosenberg JA A novel pre-clinical in vivo mouse model for malignant brain tumor growth and invasion.. J Neurooncol.

[99] Jia J, Cai Y, Wang R, Fu K, Zhao YF (2010). Overexpression of allograft inflammatory factor-1 promotes the proliferation and migration of human endothelial cells (HUV-EC-C) probably by up-regulation of basic fibroblast growth factor.. Pediatr Res.

[100] Kim DS, Han JH, Kwon HJ (2003). NF-kappaB and c-Jun-dependent regulation of macrophage inflammatory protein-2 gene expression in response to lipopolysaccharide in RAW 264.7 cells.. Mol Immunol.

[101] Seyfried TN, Shelton LM. Cancer as a metabolic disease.. Nutr Metab (Lond).

[102] Seyfried  TN, Kiebish  MA, Marsh J, Shelton  LM, Huysentruyt LC, Metabolic management of brain cancer.. Biochim Biophys Acta.

[103] Shelton LM, Huysentruyt LC, Mukherjee P, Seyfried TN. (0038). Calorie restriction as an anti-invasive therapy for malignant brain cancer in the VM mouse. ASN Neuro.

[104] Mukherjee P, Sotnikov AV, Mangian HJ, Zhou JR, Visek WJ (1999). Energy intake and prostate tumor growth, angiogenesis, and vascular endothelial growth factor expression.. J Natl Cancer Inst.

